# Mechanical characterization of PVA hydrogels’ rate-dependent response using multi-axial loading

**DOI:** 10.1371/journal.pone.0233021

**Published:** 2020-05-12

**Authors:** Wanis Nafo, Adil Al-Mayah

**Affiliations:** 1 Civil and Environmental Engineering Department, University of Waterloo, Waterloo, Ontario, Canada; 2 Mechanical and Mechatronics Engineering Department, University of Waterloo, Waterloo, Ontario, Canada; 3 Centre for Bioengineering and Biotechnology, University of Waterloo, Waterloo, Ontario, Canada; Politecnico di Milano, ITALY

## Abstract

The time-dependent properties of rubber-like synthesized and biological materials are crucial for their applications. Currently, this behavior is mainly measured using axial tensile test, compression test, or indentation. Limited studies performed on using multi-axial loading measurements of time-dependent material behavior exist in the literature. Therefore, the aim of this study is to investigate the viscoelastic response of rubber-like materials under multi-axial loading using cavity expansion and relaxation tests. The tests were performed on PVA hydrogel specimens. Three hyperelasitc models and one term Prony series were used to characterize the viscoelastic response of the hydrogels. Finite element (FE) simulations were performed to verify the validity of the calibrated material coefficients by reproducing the experimental results. The excellent agreement between the experimental, analytical and numerical data proves the capability of the cavity expansion technique to measure the time-dependent behavior of viscoelastic materials.

## Introduction

Rubber-like materials are known for their viscoelastic behavior. Elaborate characterization of this behavior is crucial to numerous applications in a variety of engineering fields and industries, in addition to the biomedical field. The time-dependent properties of these materials control their mechanical response under thermal conditions [1,2], and large deformations [3,4]. Thus, these properties have been the focus of numerous studies that utilized different mechanical characterization techniques.

In the biomedical field, studying the viscoelastic properties of biological tissues indicated that their mechanical properties change based on their pathological conditions. For instance, fibrosis was reported to change the viscoelastic behavior of liver tissues [5,6]. This change occurs at early stages of fibrosis mainly due to degeneration of fatty tissues [7]. The mechanical quantification of such diseases at early stages is very critical for treatment processes. Currently, the measurement of the mechanical properties of diseased and healthy tissues in-vivo is commonly achieved using transient elastography (TE) [8,9]. Although this technique is capable of measuring the mechanics of tissues, it is mainly used to quantify their Young’s modulus when small deformations are applied as demonstrated in [10,11]. On the other hand, TE proved to be efficient in measuring the viscous (relaxation) response of these tissues in-vivo [12].

In general, a number of theories have been developed to describe the time-dependent response of different materials. Those theories describe materials as linear viscoelastic (LVE) [13], quasi-linear viscoelastic (QVE) [14], and non-linear viscoelastic (NVE) [15,16]. The LVE description is the most common due to its simplicity in addition to the validity of the superposition principal it utilizes. Moreover, it has been thoroughly examined mathematically [17], and experimentally [18]. It is also available in commercial finite element (FE) packages. Therefore, it is the focus in this study.

The implementation of the LVE theory is based on combining the instantaneous elastic response of a given material with its viscous behavior. This behavior is commonly measured by the creep test [19], or the relaxation test [20], including in-vivo characterization in cases of biological tissues [12]. The elastic behavior is measured commonly by using axial loading and indentation [21–23]. These conventional techniques are used to measure the material response under different deformation rates. Although uniaxial loading is very common when studying the mechanics of viscoelastic materials, to our knowledge, little attention has been paid to viscoelasticity characterization using multi-axial loading. Thus, in this work, we aim to investigate the viscoelastic properties of a rubber-like material under different rates by using cavity-based multi-axial loading (pressure + equi-biaxial tension). This technique is based on inducing an expanding cavity within the structure of soft materials by using a balloon and an incompressible fluid. This technique was found to be efficient in measuring the hyperelastic properties of rubber-like materials [24,25]. Therefore, it represents a good option to investigate the rate-dependent behavior of such materials under multi-axial loading condition.

A hyperelastic material that exhibits the LVE behavior is PVA hydrogel as will be demonstrated in this study. These hydrogels are considered excellent alternatives to biological tissues due to their high hydrophobicity [26,27], biocompatibility [28], mechanical strength [29,30], physical integrity under large deformation [24,31], viscoelastic properties [32,33], thermal stability and non-toxicity [34]. Thus, they are the most suitable material for this study. Cavity expansion tests and simple shear relaxation tests are conducted to measure the instantaneous and long-term behaviors of PVA hydrogels, respectively. The data obtained from these tests will be used to calibrate the material constants of hyperelastic models (Yeoh, Arruda-Boyce and Ogden), and the Prony series viscoelastic model coefficients. Finally, FE simulations are performed to reproduce the time dependent behavior of PVA hydrogels.

## Material and methods

### Samples preparations

**Fig 1:**
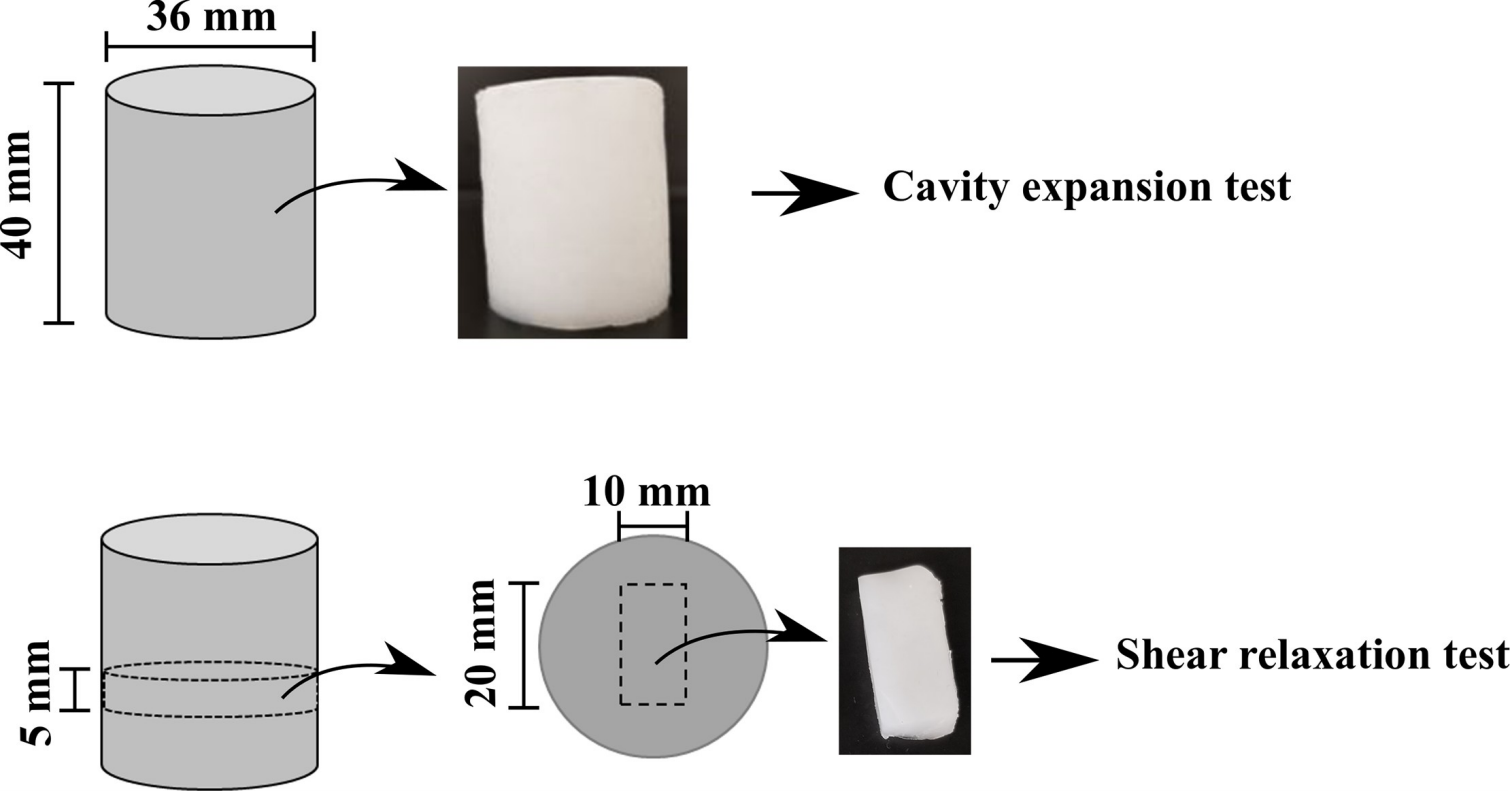
PVA hydrogels samples’ geometry. The cylindrical samples are used in the cavity test, and the cuboid samples are used in simple shear relaxation tests.

This study is based on using the cavity expansion and the relaxation tests to measure the instantaneous and time-dependent behaviors of the PVA hydrogels, respectively; thus, twelve cylindrical samples (40 mm in height and 36 mm in diameter) were prepared. The synthesis process was based on physically crosslinking PVA solution. The solution was made by mixing 99+% hydrolyzed PVA (molecular weight of 146000–186000 g.mol^-1^ with deionized water (10% w/w ratio) at 90 ^o^C by using standard flask/column combination. Thereafter, the solution was poured into cylindrical molds. The hydrogels were formed by freezing the solution at -20 ^o^C for 3 hours and then thawing at room temperature (~21 ^o^C) for 3 hours. This freeze-thaw cycle (FTC) was repeated two times. Thereafter, three gel cylinders were cut into smaller cylinders (5 mm in height) then punched in the middle to create rectangular cuboid samples with dimensions of 20 mm in length, 10 mm in width and 5 mm in thickness. Fig 1 shows the samples’ geometry for both cavity expansion and shear tests.

### Experimental setup

#### Cavity expansion test

**Fig 2:**
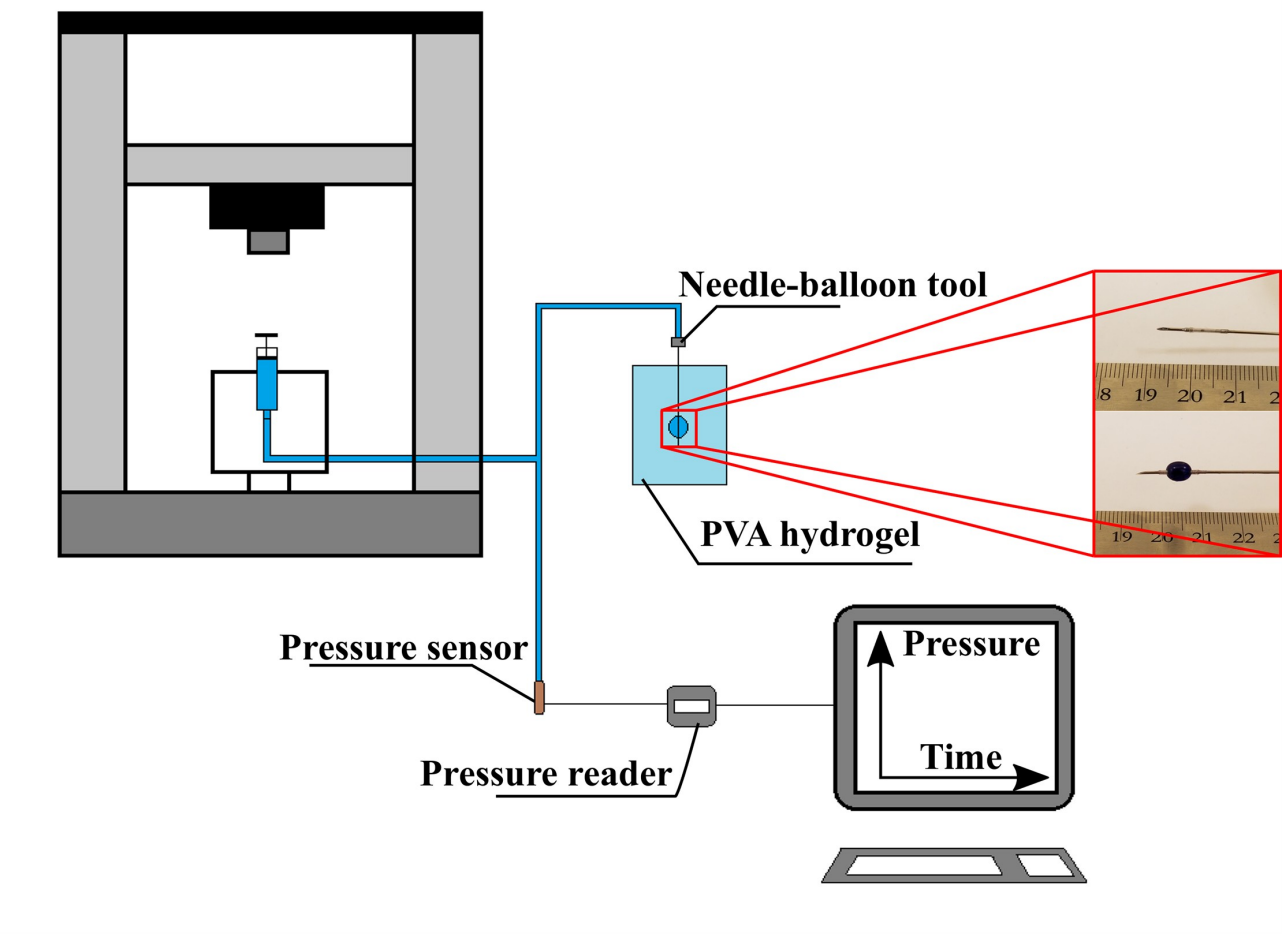
The loading set up for the cavity expansion test.

The procedure followed in [24] was adopted to perform the cavity test. However, few changes were made to the system that was used: pressure sensor (Model PRESS-S-000, PENDOTECH, USA) and pressure reader (Model PMAT-S, PENDOTECH, USA) were used to observe the pressure due to the pressurized incompressible fluid (water). In addition, an Instron machine (Model 4465; Canton, MA, USA) was used to introduce the water into the needle-balloon tool. A new design needle-balloon tool was used in this study, the balloon is silicon based with smaller dimensions and has softer mechanical behavior than the one used in the previous study. To ensure no air was entrapped inside the system, its elements (syringe, needle-balloon tool and Y-shaped tubes) were filled with water and submerged under water until they sank and assembled together. Afterwards, the tubes were connected to the pressure sensor with a water inlet and outlet, which allows depleting any remaining air in the system. Nine hydrogel samples were investigated by this test at three different deformation rates: 5 μl/s, 20 μl/s and 50 μl/s. A schematic diagram for the system is shown in Fig 2. In addition, the balloon response was evaluated separately using the Instron machine.

#### Relaxation test

**Fig 3:**
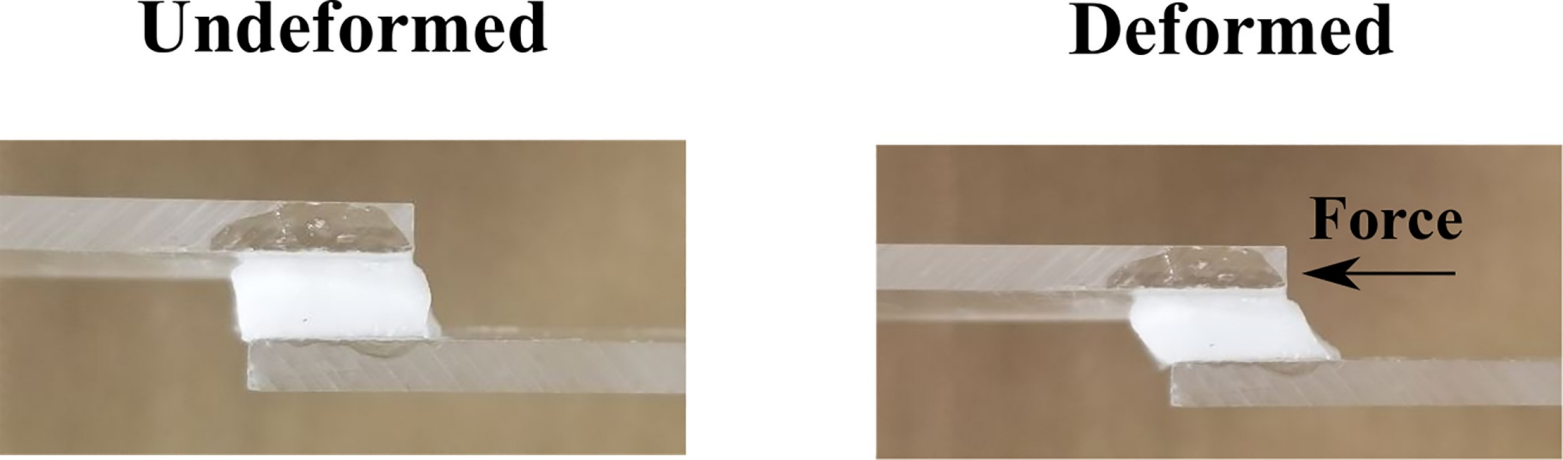
The loading configuration for the shear relaxation tests. The cuboid was subjected to 50% shear strain and left to relax for 300 seconds.

This test was performed on the gel cuboids; they were subjected to simple shear relaxation test. Acrylic platens (50 mm × 20 mm × 2.5 mm) were machined with spacers glued to the platens to ensure a good alignment as well as to avoid overstressing of the gel specimens. The top and the bottom surfaces of the cuboid specimens were placed and glued between the platens with a thin layer of a fast-acting adhesive. The assembly was then mounted on the Instron machine and loaded at a rate of 0.1 s^-1^. The relaxation test was performed at various shear strains: 10%, 20%, 30%, 40%, 50%, 60%, 70% and 80% to investigate the behavior of the hydrogels in a step-like strain at various magnitudes. Fig 3 shows the loading configuration for the simple shear relaxation test.

## Analytical framework

In this work, the time-dependency of the PVA hydrogel behavior is modelled as LVE. This description is considered accurate for the tested gels as will be shown in section 4. A general description of the LVE material stress in large deformations at time (t) can be expressed as reported in [35]

$$\sigma (t)=\sigma_{0}(\varepsilon (t))-\int_{0}^{t}\dot{g}(t-\tau )\sigma_{0}(\varepsilon (t))d\tau$$ (1)

The first term represents the hyperelastic stress function (σ_h.el_). The second term represents Prony series expression of stress relaxation function (σ_v.el_).

At time t+Δt, and by using “N” number of terms in the Prony series, Eq 1 can be expressed as

$$\sigma (t+\Delta t)=\sigma_{h.\mathrm{el}}(t+\Delta t)‐\sum_{i=1}^{N}\sigma_{v,\mathrm{el}}^{i}(t+\Delta t)$$ (2)

σ_h.el_ (t+Δt) can be evaluated by using material parameters of a calibrated strain energy function (SEF), and, while $\sigma_{v.el}^{i}$ (t+Δt) can be expressed as

$$\sigma_{v.\mathrm{el}}^{i}(t+\Delta t)=e^{-\frac{\Delta t}{\tau_{i}}}\cdot \sigma_{v.\mathrm{el}}^{i}(t)+g_{i}\cdot \sigma_{h.\mathrm{el}}(t)\left[1-e^{-\frac{\Delta t}{\tau_{i}}}\right]+g_{i}\frac{\Delta \sigma_{h.\mathrm{el}}}{\Delta t}\left[\left(\Delta t-\tau_{i}\right)+\tau_{i}e^{-\frac{\Delta t}{\tau_{i}}}\right]$$ (3)

Where g_i_ and τ_i_ are the relaxation parameters. See [35] for full derivation.

A number of strain energy functions (SEFs) are available in literature. In this work, we focus only on three SEFs, namely: Yeoh, Ogden and Arruda-Boyce. These models are available in most of the commercial FE software packages; in addition, they proved to be efficient in capturing the non-linear elastic response of rubber-like materials subjected to uniaxial loading, biaxial loading [36], and cavitation [24]. Yeoh, Ogden, and Arruda-Boyce models are expressed, respectively, as

$$W=\sum_{i=}^{N=3}C_{i0}(I_{1}-3{)}^{i}$$ (4)

$$W=\sum_{i=1}^{N=3}\frac{2\mu_{i}}{\alpha_{i}^{2}}\left(\lambda_{1}^{\alpha_{i}}+\lambda_{2}^{\alpha_{i}}+\lambda_{3}^{\alpha_{i}}-3\right)$$ (5)

$$W=\mu \sum_{i=1}^{5}\frac{C_{i}}{\lambda_{m}^{2i-2}}\left(I_{1}^{i}-3^{i}\right)$$ (6)

Where μ_*i*_, α_i_, C_i0_ and C_i_ are material constants; λ_m_ is the extensibility limiter.

In the cavity expansion test, the pressure applied on the cavity wall generates radial stress normal to the wall and hoop circumferential stresses along the wall. These stresses can be expressed in Cauchy hyperelastic form as

$$\sigma_{r}=2\left[W_{1}\lambda^{‐4}+2W_{2}\lambda^{‐2}\right]$$ (7)

$$\sigma_{\theta }=\sigma_{\varphi }{}_{}=2\left[W_{1}\lambda^{2}+W_{2}\left(\lambda^{4}+\lambda^{‐2}\right)\right]$$ (8)

Where W_1_ and W_2_ are the SEF derivatives with respect to I_1_ and I_2_, respectively. Eq 8 can be used to calculate σ_h.el_ (t+Δt).

For material calibration using Eqs 4, 5 and 6 in the cavity test, we can use a tangential relationship that relates the radial stress (σ_r_) and the hoop deformation (λ), see [24,37] for derivation

$$\frac{{\mathrm{d\sigma}}_{r}}{\mathrm{d\lambda}}=\frac{\hat{W}}{\lambda^{3}-1}$$ (9)

Where $\hat{W}$ is the derivative of W with respect to λ. The hyperelastic term of Eq 2 can be calibrated by substituting the derivatives of Eqs 4, 5 and 6 into Eq 9.

## Results

### Cavity expansion tests

**Fig 4:**
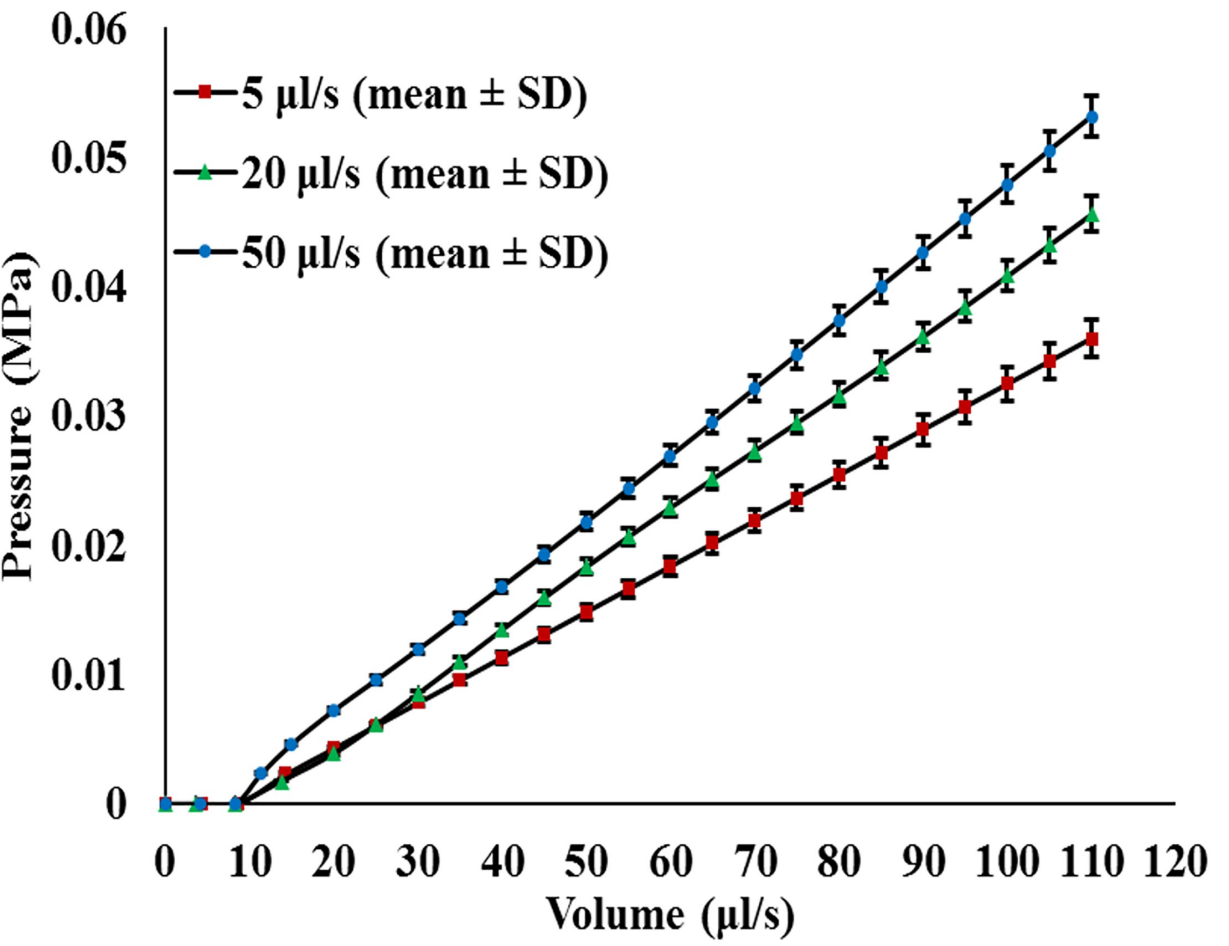
Cavity tests. PVA hydrogel specimens were loaded up to 110 μl injection volume at loading velocities of 5 μl/s, 20 μl/s and 50 μl/s.

The cavity tests were performed on cylindrical specimens at each injection rate ($\dot{V}$) of 5 μl/s, 20 μl/s and 50 μl/s up to a volume of 110 μl in order to investigate the behavior of the gels at particular loading rates, as shown in Fig 4. The pressure (MPa) and time (s) data were measured directly by the system. The volume data shown in Fig 4 were calculated through multiplying the time data by their corresponding injection rate (t **ˑ** $\dot{V}$). It was observed that, at the initial stage of the balloon inflation, the gels did not show any resistance for time periods of 1.96 s, 0.45 s and 0.16 s at injection rates of 5 μl/s, 20 μl/s and 50 μl/s, respectively. Multiplying these short periods of times by their corresponding volume rates will result in initial volumes (V_i_) of 8.635 μl, 8.23 μl and 8.24 μl, respectively. The lack of resistance to these volumes can be due to the displacement of the channel wall created by the needle during the insertion process, which relieves any constraints against balloon inflation at early stages. Afterwards, the gels start to resist deformation as the balloon inflates spherically. During the test, the hydrogels were internally deformed to a volume of 110 μl. At this volume, the balloon material started to participate in resisting the inflation. Thus, to avoid the effect of the balloon’s silicon material, all tests stopped at 110 μl. The balloon response was investigated separately at the lowest rate (5 μl/s) by the Instron machine, using the built-in linear variable differential transformer (LVDT) and load cell, see S1 Fig. The effect of the friction between the rubber-stopper and the syringe plunger was also investigated, see S2 Fig

.

### Relaxation test

**Fig 5:**
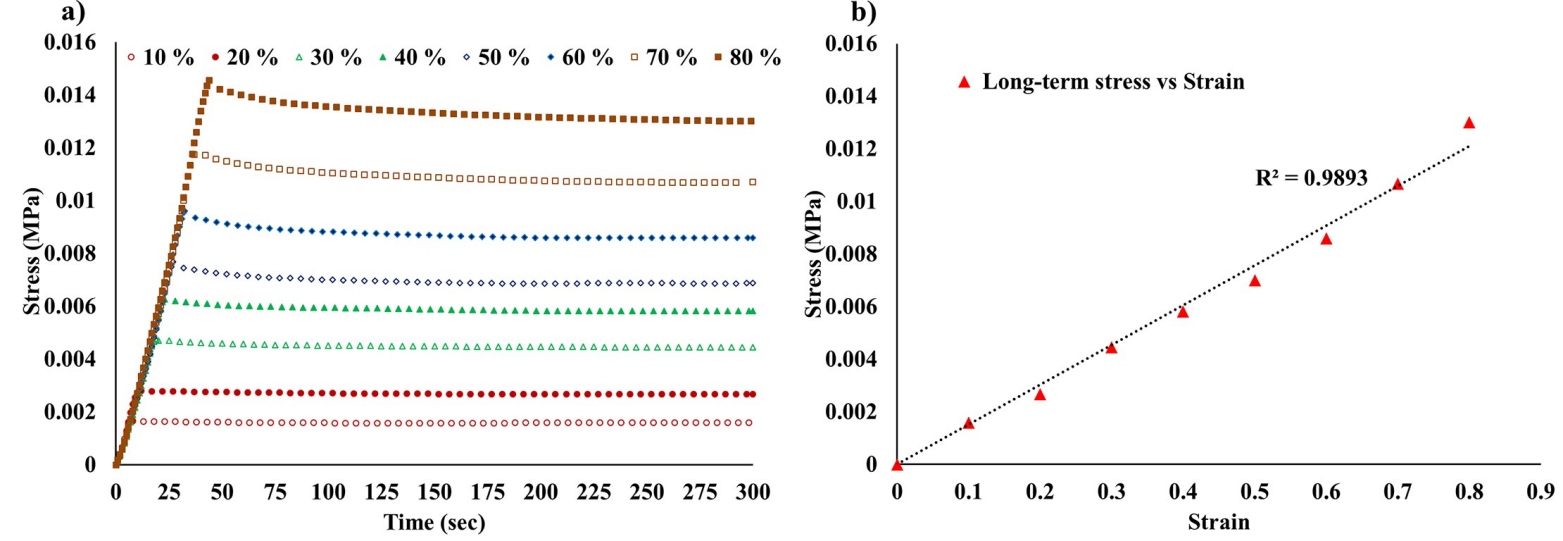
Simple shear relaxation test. a) Long-term shear stress at various strain magnitudes; b) the long-term shear stress-strain relationship. The high value of R^2^ (0.9893) is an indication to the validity of considering the material as linear viscoelastic.

The cuboid specimens were loaded at various shear strain levels (10% - 80%) and held for 300 seconds to record the relaxation force as stresses showed no tangible relaxation after 300 seconds of loading. The force data was then converted into stress form using the cuboid dimensions, as shown is Fig 5A. The long-term shear stresses and their correspondent strains were plotted, and it was found that the gels exhibited a linear long-term behavior, see Fig 5B, which indicates the validity of the LVE assumption. It is worth mentioning that stress-strain data in the simple shear test represented plane stress-plane strain behavior, i.e., the initial area (20 mm × 10 mm) that was used to calculate the shear test from the force data remains unchanged after loading the specimens.

### Calibration of SEFs and Prony series

The experimental data obtained from the cavity expansion tests were in pressure (P)–volume (V). The deformation term (V) was converted into a hoop stretch term (λ), which was calculated as $\frac{r}{R}$. “R” values were found to be 1.399 mm, 1.382 mm and 1.383 mm at injection rates of 5 μl/s, 20 μl/s and 50 μl/s, respectively. These magnitudes were calculated using the concept of Equivalent Volume Diameter (EVD) [38] as follows:

$$R=\frac{\sqrt[{3}]{\frac{6V_{i}}{\pi }}}{2}$$ (10)

V_i_ can be evaluated as

$$V_{i}=V_{n‐b}+V_{p}$$ (11)

Where V_n-b_ is cylindrical volume (5 mm in length and 0.85 mm in diameter) of the balloon region before inflation; V_p_ are the volumes of water introduced into the balloon, 8.635 μl, 8.23 μl and 8.24 μl (calculated in the Results section), before the hydrogel started to resist balloon inflation at each of injection rates, 5 μl/s, 20 μl/s, 50 μl/s, respectively.

The magnitude of “r” was calculated as

$$r=\sqrt[{3}]{\frac{3\left(V_{i}+V_{\mathrm{app}}\right)}{4\pi }}$$ (12)

Where V_app_ is the injected volumes of water during the cavity expansion test.

**Fig 6:**
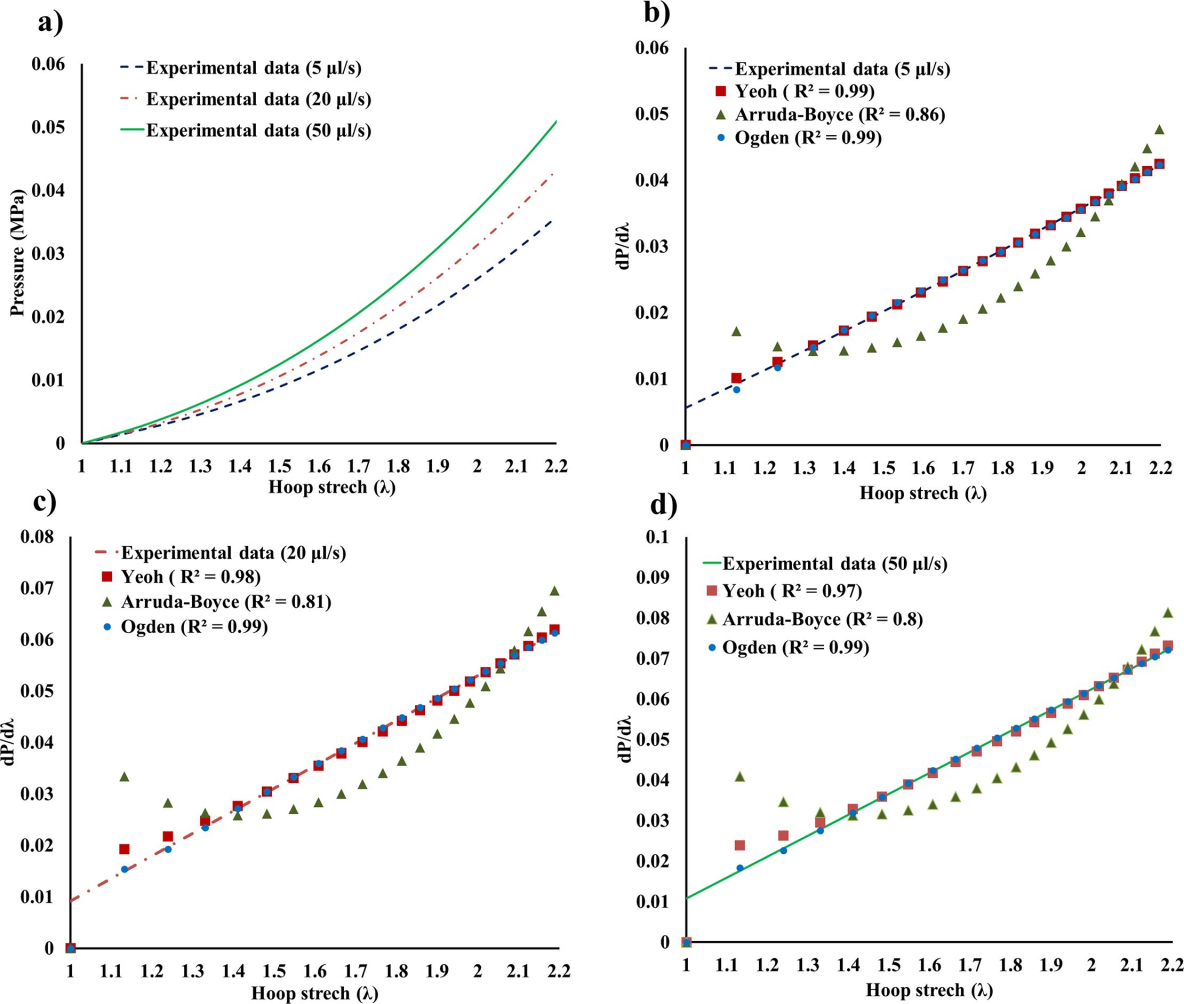
Experimental data. a) Experimental data Pressure vs stretch (P–λ). Segments b, c and d show the agreement between the SEFs and the instantaneous rate of change derived from the experimental data at injection rates of 5 μl/s, 20 μl/s and 50 μl/s, respectively.

Fig 6A shows the experimental data in the form of P–λ of the three injection rates. The instantaneous rate of change of the three curves was calculated. Before the commencement of the calibration process, it is worth mentioning that due the local nature of the cavity expansion test, Eq 9 can be expressed as

$$\frac{\mathrm{dP}}{\mathrm{d\lambda}}=\frac{\hat{W}}{\lambda^{3}-1}$$ (13)

This indicates that the radial stress at the cavity wall, where the material parameters are calculated, is equivalent to the applied pressure.

The resulting theoretical curves exhibited strong agreement with experimental data. Arruda-Boyce model showed the least fit, yet it is still valid, (Fig 6B, 6C and 6D). The material parameters are summarized in Table 1.

**Table 1:** Material parameters obtained after calibrating the SEFs model to experimental data (C_i_ and μ_i_ are in MPa).

μl/s	Yeoh			Arruda-Boyce		Ogden					
	C_10_	C_20_	C_30_	μ	λ_m_	μ_1_	μ_2_	μ_3_	α_1_	α_2_	α_3_
5	1.3e-03	1.798e-03	1.594e-04	2.5e-03	1.127	0.0514	0.01106	-0.06	0.0378	6.126	0.679
20	2.75e-03	2.57e-03	2.35e-04	5.7e-03	1.203	0.016	0.0409	-0.0515	0.0624	5.147	2.833
50	3.5e-03	2.9e-03	3e-04	7.1e-03	1.215	0.0339	0.0383	-0.065	0.5402	5.317	2.059

The initial shear moduli of the PVA hydrogels can also be estimated from the calibrated material parameters at each deformation rate. These moduli are summarized in Table 2.

**Table 2:** Initial shear moduli estimated by the SEFs at the three deformation rates.

μl/s	μ_Yeoh_ = 2C_10_ (MPa)	μ _`Arruda-Boyce_ = μ (MPa)	μ _Ogden_ = ∑μ_*i*_ (MPa)
5	2.6e-03	2.5e-03	2.46e-03
20	5.5e-03	5.7e-03	5.4e-03
50	7e-03	7.1e-03	7.2e-03

**Fig 7:**
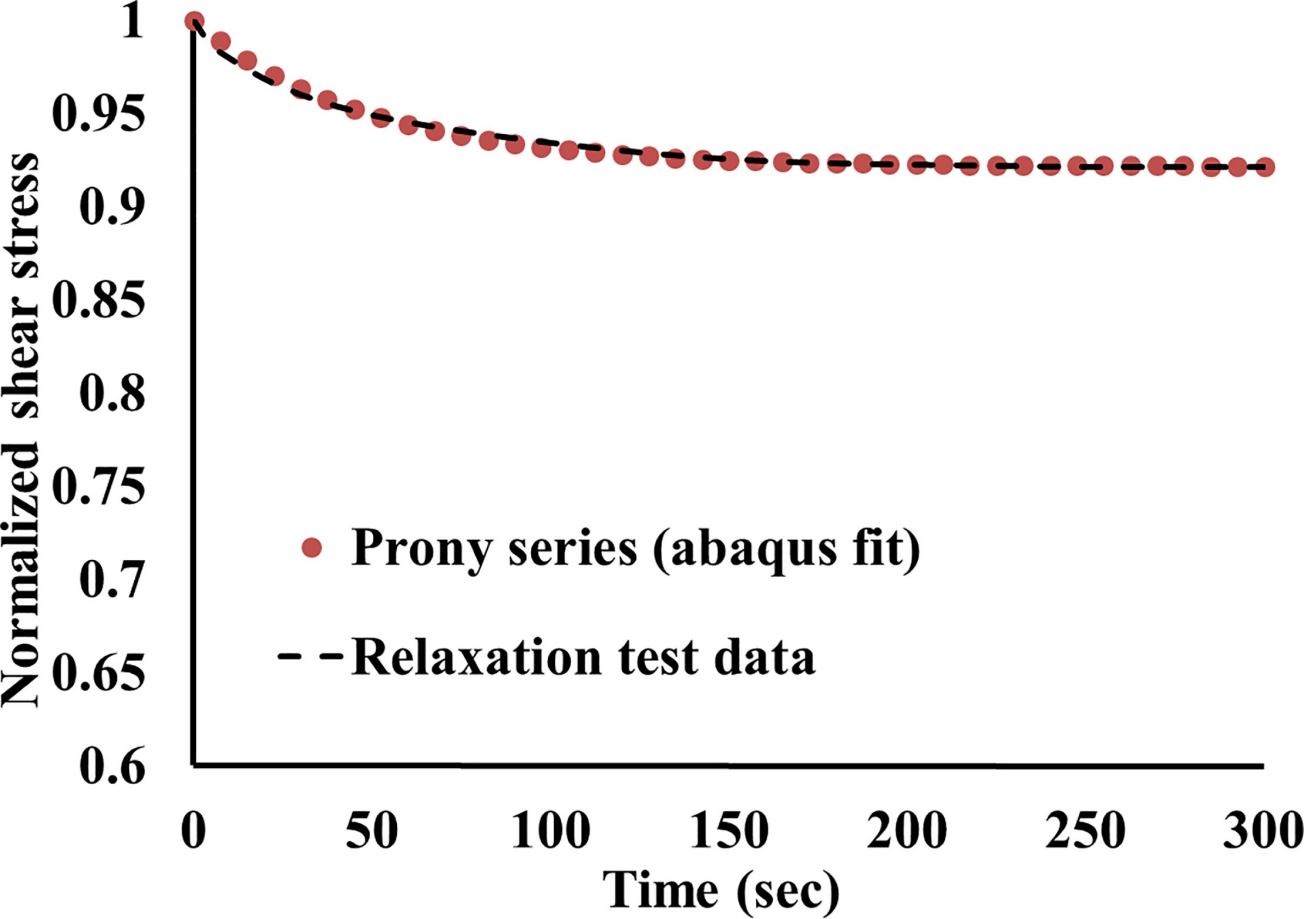
Long term gel response. Comparison between the relaxation test data and the Prony series function.

In this work, Abaqus^®^ software package was used to evaluate the Prony series coefficient using its built-in calibration tool. The long-term shear stress data, shown in Fig 5, was converted into shear moduli data, and then normalized and input in Abaqus^®^ to run the calibration process. One term Prony series provided an excellent fit to the relaxation response of the hydrogel, see Fig 7. The Prony series coefficients were g = 7.837e-02 and τ = 47.845 s.

## Finite element analysis

Most of the reported work on the viscoelasticity of PVA hydrogels was describing the material as QLV [39,40]. Thus, it was necessary to validate the predictable behavior of the LVE description adopted in this study by using numerical simulations, an analytical solution, and measured experimental data.

In order to reproduce the experimental results, FE simulations were performed using Abaqus^®^ by implementing the model described in [24], and the material parameters obtained in the previous section. A mass density of 1030 kg/m^3^ (±65 kg/m^3^) was used for the model’s elements (CAX4H). This magnitude was estimated by using Archimedes principal. The observed pressure during the experiments was applied in the FE simulations.

**Fig 8:**
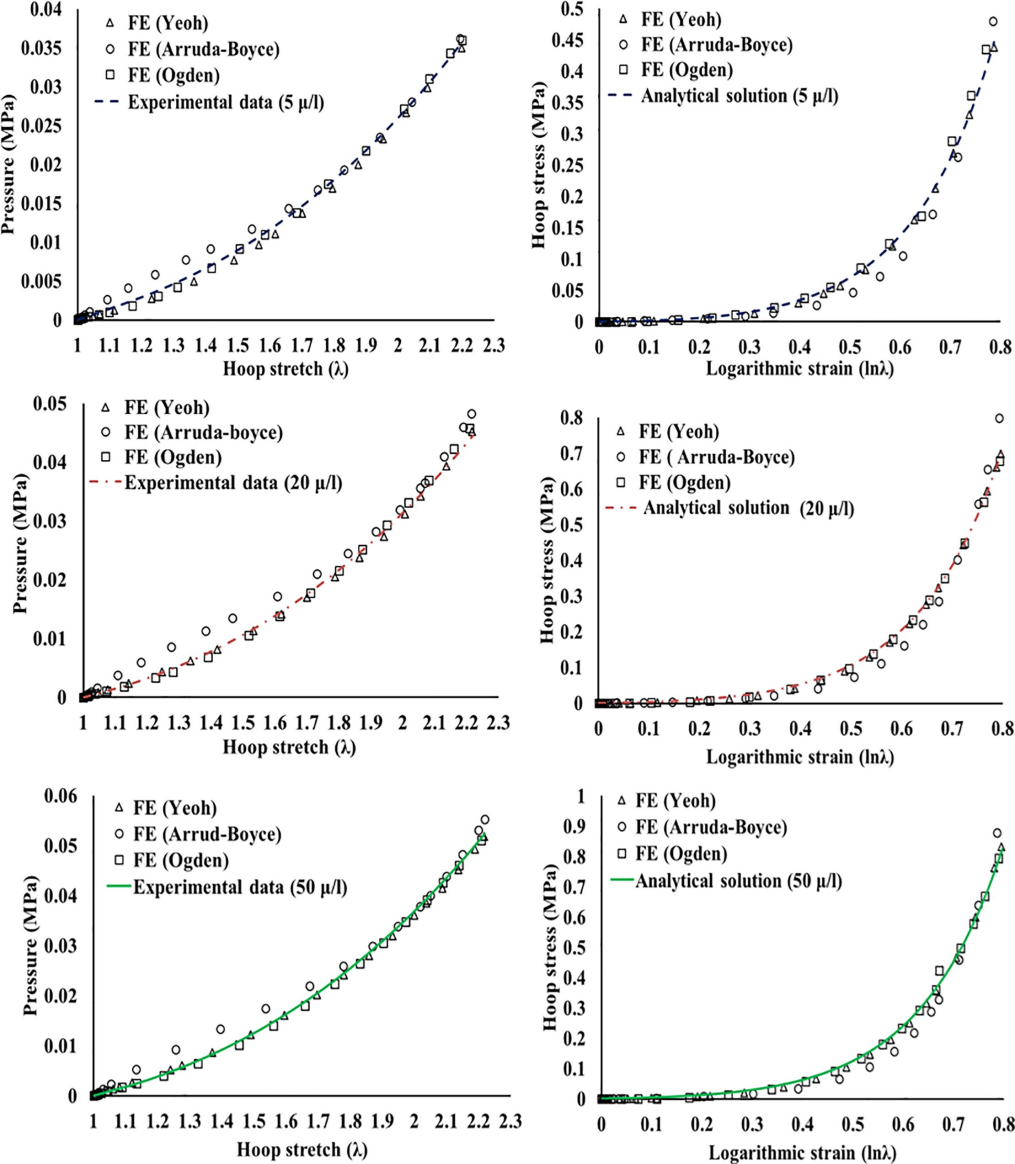
Experimental, numerical, and analytical results. The numerical results compared against the experimental pressure and the analytical hoop stress at each deformation rate.

The FE simulations predicted numerical pressure (radial stresses at the cavity wall) that was in a good agreement with experimental pressure. In addition, the simulations provided numerical hoop stresses that were matching their analytical counterparts calculated using Eq 2, see Fig 8. This equation can be solved using Matlab^®^, see S1 Appendix for an example solution using Yeoh model for 50 μl/s data.

The numerical results showed an excellent agreement with the experimental data. In addition, they predicted numerical hoop stresses that are in robust agreement with the analytical hoop stresses. These two agreements at each of the deformation rates are good indications to the strong validity of the calibrated material parameters of the implemented SEFs.

The data obtained from the cavity tests were experimental P-λ and analytical σ_θ_—ε_θ_. The SEFs’ parameters, used to fit experimental data as well as to predict the σ_θ_—ε_θ_ data analytically, were verified by using FE. The root mean square (RMS) error was calculated to evaluate the difference between the realistic data (experimental and analytical) and the reproduced data (numerical). The error values are summarized in Table 3

$$\mathrm{RMS}\;\mathrm{error}=\frac{1}{N}\sqrt{\sum_{i=1}^{N}\frac{{\mathrm{Load}}_{\mathrm{experimental}\;\mathrm{or}\;\mathrm{analtical}}-{\mathrm{load}}_{\mathrm{FE}}}{{\mathrm{Load}}_{\exp}}}$$ (14)

**Table 3:** RMS error values for each of the SEFs.

	Yeoh model		Arruda-Boyce model		Ogden model	
μl/s	Pressure data	Hoop stress data	Pressure data	Hoop stress data	Pressure data	Hoop stress data
5	3.1%	4.1%	7.7%	9.5%	3.5%	2.4%
20	1.9%	3.8%	9.8%	8.6%	2.4%	3.6%
50	2%	4.1%	9.1%	9%	3.4%	3.4%

## Discussion

The characterization of PVA hydrogels using cavity expansion at different deformation rates allows for measuring the rate-dependent response of rubber-like materials in multi-axial loading. In this research, the properties of PVA hydrogels subjected to cavitation loads have been characterized up to 80% hoop logarithmic strain at deformation rates of 5 μl/s, 20 μl/s and 50 μl/s. Force relaxation experiments in simple shear were also performed at various strain magnitudes (10% - 80%).

The parameters of the three SEFs were calibrated through Eq 9, while the time-dependent Prony coefficients were calibrated using Abaqus’ calibration tool. Overall, the good agreement between the numerical, experimental and analytical results indicates that the three SEFs are suitable to model the viscoelastic behavior of the PVA hydrogels up to 80% strain.

The initial shear moduli of the PVA hydrogels were estimated by the SEFs to be ≈ 2.5 kPa, 5.5 kPa and 7 kPa at deformation rates of 5 μl/s, 20 μl/s and 50 μl/s, respectively. While the three models predicted similar initial shear moduli at each deformation rate, the overall performance of Arruda-Boyce model in data fitting was the least sufficient. This is due to its limited number of parameters unlike the other two phenomenological models. However, Arruda-Boyce model has the capacity to predict the material response in other forms of loading using the same material parameters due to its micromechanical nature (use of eight Langevin chains network) [41,42], an advantage that does not exist in other empirical models such as Yeoh and Ogden.

In this study, the PVA hydrogels were considered incompressible. This assumption was based on the very low compressibility nature of this type of gels. Nafo and Al-Mayah [24] reported that there is no significant difference between the compressible and incompressible assumptions in simulating the behavior of PVA hydrogels subjected to cavity loads. In addition, Chen et al [43] reported a relatively high Poisson’s ratio for this type of hydrogels (≈0.5); thus, we considered the gels as incompressible materials in this study. It is also worth mentioning that the volumetric behavior of most rubber-like materials has very little or no time-dependency [44]. Therefore, only the relaxation parameters (g_i_ and _τ_) were calibrated and considered for the viscous response of the hydrogels.

To investigate the syringe-balloon response as well as the injection system at various injection rates, we also collected data from the machine’s load cell. The friction force between the rubber-stopper and the inner wall of the plunger increased when the injection velocity was increased as shown in S2 Fig. Overall, the friction response is divided into two stages, static and dynamic. The dynamic stage seems very steady when the injection velocity was low. However, when the injection velocity was increased, the force data exhibited relatively volatile behaviors. The two friction phases observed by the load cell makes it difficult to estimate V_p_, particularly, at high velocities, which may lead to fallible calculation of “λ” and inaccurate characterization of material properties. Therefore, observing the pressure from an independent source (pressurized water that flows into the system) will result in measurements that are more accurate. There are limited data, to authors’ knowledge, available on testing the viscoelastic response of PVA hydrogels under multi-axial loading condition; however, the test proved to be capable of measuring the rate-dependent response of the hydrogels. Attributed to its multi-axial nature and simplicity, the technique has the potential to provide enough information about in-vivo viscoelastic behavior of biological tissues in different loading axes, which will be addressed in a different study.

There are a number of models to describe linear viscoelasticity [45,46]. However, the most commonly used model is the generalized Maxwell model. This model is usually represented by a Prony series [47], which is used to fit the experimental data to evaluate the discrete times (**τ**_i_) and the long term shear moduli (**g**_i_). Although this representation is feasible and the fitting process is relatively simple, Prony series is prone to challenges related to the coefficients evaluation process, particularly, when the fitting process renders negative coefficients, which result in oscillations in the modelled material behavior [48]. However, this challenge can be addressed using several fitting approaches that have been developed over the past few decades [49–51]. On the other hand, the use of Prony series is efficient analytically and numerically as shown in this study, if the coefficients were determined correctly. This is mainly because Prony series permits analytical integration of the relaxation equations.

## Conclusions

Measuring the rate-dependent response of rubber-like materials commonly performed using uniaxial loading. This investigation introduced an alternative that allows for multi-axial measurement to the viscoelastic properties of these material. The following conclusions can be made from this study:

- The hydrogel specimens were loaded up to 80% hoop strain, and the corresponding hoop stresses were 0.44 MPa, 0.7 MPa and 0.82 MPa at strain rates of 5 μl/s, 20 μl/s and 50 μl/s, respectively. At the same rates, the observed pressure magnitudes were 0.035 MPa, 0.044 MPa and 0.052 MPa, respectively.
- Yeoh and Ogden models provided good fit to the experimental data (R^2^ = 0.97 to 0.99), while Arruda-Boyce model provided the least fit to the experimental data (R^2^ = 0.8 to 0.86). However, the three models provided similar initial shear moduli in each of the deformation rates.
- The agreement between the experimental, analytical and numerical data indicates that the cavity expansion test is capable of measuring the rate-dependent response of rubber-like materials.
- The relaxation coefficients of Prony series can be used with the material parameters of the three SEFs to perform linear viscoelastic analysis for PVA hydrogels in numerical solvers such as Abaqus^®^.
- The cavity expansion technique proved to be capable of measuring the rate-dependent properties of rubber-like materials. Thus, it has the potential to measure the viscoelastic properties of biological tissues.

## Supporting information

S1 Appendix Matlab file name: LVE_Large_deformation_Yeoh_model. (PDF)

S1 Fig Balloon response investigation. a) Acrylic frame used in the cavity expansion test, and the balloon configuration during the inflation process. b) Balloon response. The loading procedure was based on applying forces on the syringe plunger by using the Instron machine. A custom-made acrylic frame was made to hold the syringe during the injection process, see S1A Fig. At the lowest rate (5 μl/s), the balloon material showed no contribution in resisting the inflation. However, at a volume of 110 μl, the force data showed hardening due to balloon material participation in resisting inflation.
(TIF)

S2 Fig Friction investigation. The friction response under the three volume rates used in the cavity expansion test: the overall response starts with instantaneous increase in the friction resistance (static friction), followed by a drop and continuous resistance (dynamic friction). The dynamic friction response at the lowest rate is steady, and volatile at higher injection velocities.
(TIF)

S1 Data (XLSX)
