# Supplementary material for: Mechanical characterization of PVA hydrogels’ rate-dependent response using multi-axial loading
Source: PLoS One. 2020 May 12;15(5):e0233021. doi: 10.1371/journal.pone.0233021 (PMC7217472; doi:10.1371/journal.pone.0233021)
Supplement: S1 Appendix — (PDF) [file pone.0233021.s001.pdf]

Appendix

Matlab File Name: LVE_Large_deformation_Yeoh_model

```
N = 100;
time = linspace(0,1,N);
strain1 = linspace(0,0.8,N);
strain = strain1;
params = [3.5e-03,2.9e-03,3e-04,7.837e-02,47.845];%[C10,C20,C30,g,tau]
stress = LVE_uniax(time, strain, params);
figure(1)
plot(strain, stress, '.-b')
xlabel('True strain')
ylabel('True stress (MPa)')
legend('LVE','Location','southeast')

%% Yeoh model. Incompressible hyperelastic hoop stress.
function st_Yh = Yh(strain, params)
C10 = params(1);
C20 = params(2);
C30 = params(3);
lam = exp(strain);
st_Yh = (C10 + 2*C20*(2*lam^2 + lam^-4 - 3) + 3*C30*...
   (2*lam^2 + lam^-4 - 3)^2)*2*(lam^2 - lam^-4);
end

%% Linear viscoelasticity.
function stress = LVE_uniax(time, strain, params)
stress = zeros(1,length(time));
g = params(4);
tau = params(5);
stressV = zeros(1,length(g));
stressH0 = Yh(strain(1), params);
for i=2:length(time)
   stressH1 = Yh(strain(i), params);
   dstressH = stressH1 - stressH0;
   dt = time(i)-time(i-1);
   stress(i) = stressH1;
   for j = 1:length(g)
        stressV(j) = exp(-dt/tau(j))*stressV(j)+...
            g(j)*stressH0*(1-exp(-dt/tau(j)))+...
            g(j)*dstressH/dt*(dt-tau(j)+tau(j)*exp(-dt/tau(j)));
        stress(i) = stress(i)-stressV(j);
   end
   stressH0 = stressH1;
end
end
```
