# Supplementary figures and images for: Mechanical characterization of PVA hydrogels’ rate-dependent response using multi-axial loading
Source: PLoS One. 2020 May 12;15(5):e0233021. doi: 10.1371/journal.pone.0233021 (PMC7217472; doi:10.1371/journal.pone.0233021)

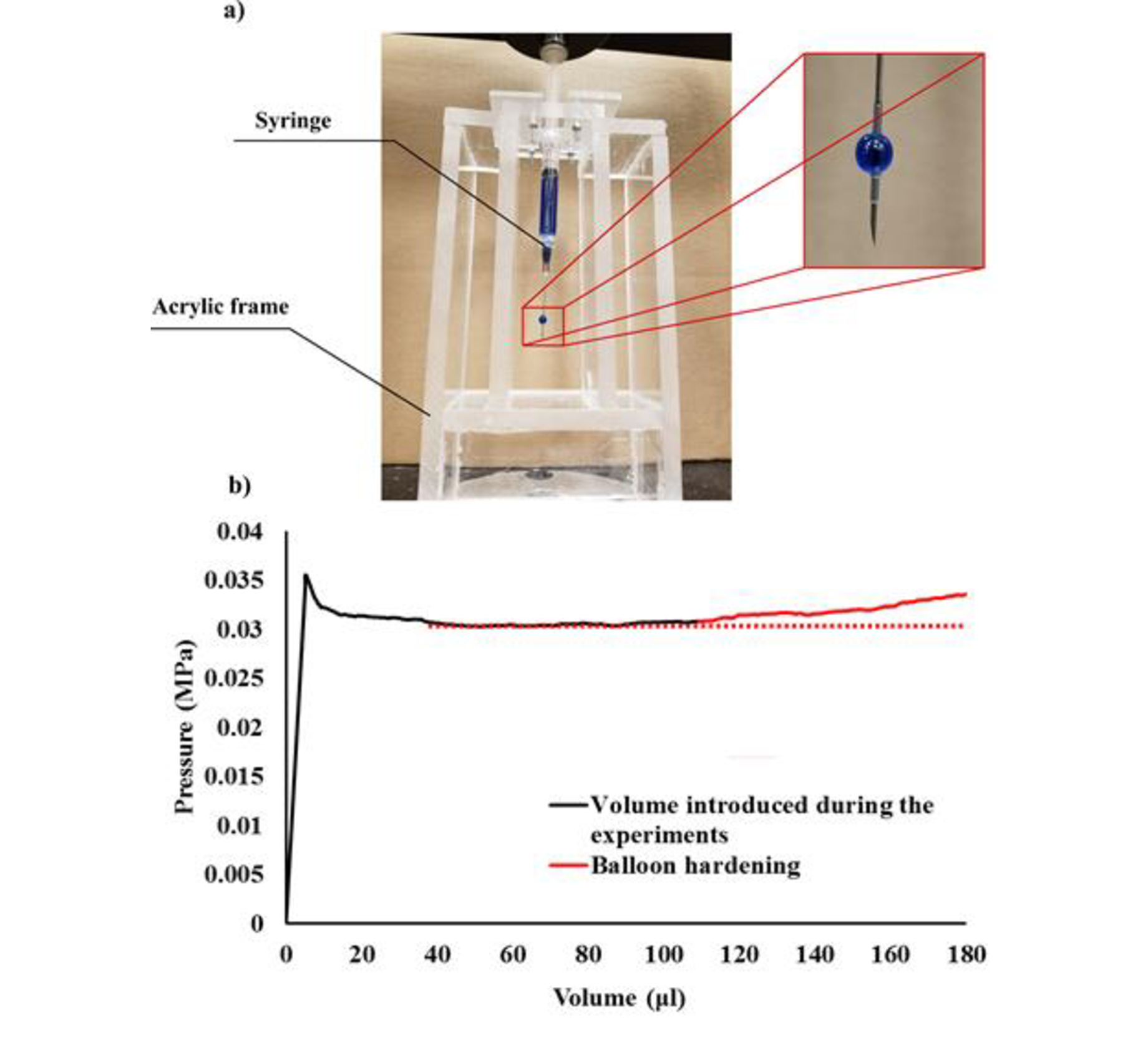

Supplement: S1 Fig — a) Acrylic frame used in the cavity expansion test, and the balloon configuration during the inflation process. b) Balloon response. The loading procedure was based on applying forces on the syringe plunger by using the Instron machine. A custom-made acrylic frame was made to hold the syringe during the injection process, see S1A Fig. At the lowest rate (5 μl/s), the balloon material showed no contribution in resisting the inflation. However, at a volume of 110 μl, the force data showed hardening due to balloon material participation in resisting inflation. (TIF) [file pone.0233021.s002.tif]

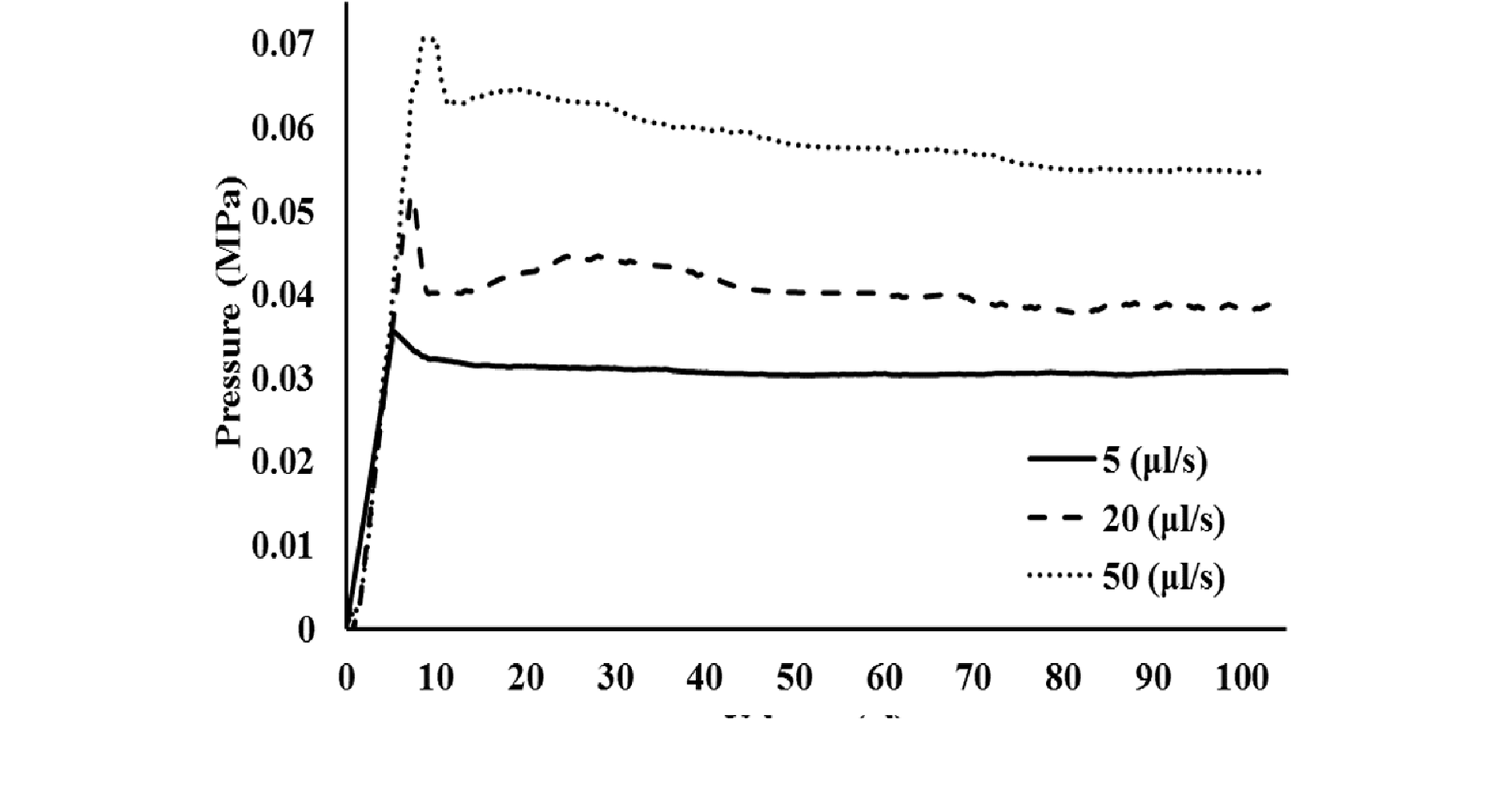

Supplement: S2 Fig — The friction response under the three volume rates used in the cavity expansion test: the overall response starts with instantaneous increase in the friction resistance (static friction), followed by a drop and continuous resistance (dynamic friction). The dynamic friction response at the lowest rate is steady, and volatile at higher injection velocities. (TIF) [file pone.0233021.s003.tif]
